# MiRNA Expression in Psoriatic Skin: Reciprocal Regulation of hsa-miR-99a and IGF-1R

**DOI:** 10.1371/journal.pone.0020916

**Published:** 2011-06-07

**Authors:** Galya Lerman, Camila Avivi, Corine Mardoukh, Aviv Barzilai, Ariel Tessone, Ben Gradus, Felix Pavlotsky, Iris Barshack, Sylvie Polak-Charcon, Arie Orenstein, Eran Hornstein, Yechezkel Sidi, Dror Avni

**Affiliations:** 1 Laboratory of Molecular Cell Biology, Center for Cancer Research and Department of Internal Medicine C , Sheba Medical Center, Tel Hashomer, Israel; 2 Sackler School of Medicine, Tel Aviv University, Tel Aviv, Israel; 3 Institute of Pathology Sheba Medical Center, Tel Hashomer, Israel; 4 Department of Dermatology Sheba Medical Center, Tel Hashomer, Israel; 5 Department of Plastic and Reconstructive Surgery and Burns Sheba Medical Center, Tel Hashomer, Israel; 6 Department of Molecular Genetics Weizmann Institute of Science, Rehovot, Israel; Istituto Dermopatico dell'Immacolata, Italy

## Abstract

**Background:**

Psoriasis is a complex disease at the cellular, genomic and genetic levels. The role of microRNAs in skin development was shown in a keratinocyte-specific Dicer knockout mouse model. Considering that two main characteristics of psoriasis are keratinocytes hyperproliferation and abnormal skin differentiation, we hypothesized that aberrant microRNA expression contributes to the psoriatic phenotype. Here, we describe the differential expression of miRNAs in psoriatic involved and uninvolved skin as compared to normal skin, revealing an additional aspect of this complex disorder.

**Methodology/Principal Findings:**

Expression arrays were used to compare microRNA expression in normal skin versus psoriatic involved and uninvolved skin. Fourteen differentially expressed microRNAs were identified, including hsa-miR-99a, hsa-miR-150, hsa-miR-423 and hsa-miR-197. The expression of these microRNAs was reevaluated by qPCR. IGF-1R, which is involved in skin development and the pathogenesis of psoriasis, is a predicted target of hsa-miR-99a. In an *in situ* hybridization assay, we found that IGF-1R and miR-99a are reciprocally expressed in the epidermis. Using a reporter assay, we found that IGF-1R is targeted by hsa-miR-99a. Moreover, over expression of miR-99a in primary keratinocytes down-regulates the expression of the endogenous IGF-1R protein. Over expression of miR-99a also inhibits keratinocyte proliferation and increases Keratin 10 expression. These findings suggest that overexpression of hsa-miR-99a in keratinocytes drives them towards differentiation. In primary keratinocytes grown in high Ca^++^, miR-99a expression increases over time. Finally, we found that IGF1 increases the expression of miR-99a.

**Conclusions/Significance:**

We identified several microRNAs that are expressed differentially in normal and psoriatic skin. One of these miRNAs is miR-99a that regulates the expression of IGF-1R. Moreover, miR-99a seems to play a role in the differentiation of keratinocytes. We suggest that miR-99a is one of the regulators of the IGF-1R signaling pathway in keratinocytes. Activation of IGF1 signaling results in elevation of miR-99a which represses the expression of IGF-1R.

## Introduction

Psoriasis is a very common chronic inflammatory skin disorder with an estimated prevalence of 2%. Despite the accessibility, frequency and persistence of this complex disease that involves different cell types and many genes, puzzling questions remain unanswered [1]. Perhaps the most obvious is the lack of major genetic susceptibility determinants, although several psoriatic associated genes have been found (Reviewed in [2]). Susceptibility to psoriasis has been mapped to loci on several chromosomes [3], [4], [5], [6], [7], [8], [9]. This multigenic disease is characterized by abnormally increased keratinocyte proliferation, abnormal differentiation of the epidermis and systemic and local inflammation, which result in the formation of chronic erythematous and scaly lesions [1], [10], [11]. Psoriatic epidermal hyper proliferation is characterized by over representation of basal keratinocytes, increased number of mitoses and their presence above the basal layer, evenly thickened epidermis with persistence of cell nuclei in the upper cornified layer, and loss of the granular layer. Keratinocyte transit time through the epidermis is accelerated 10-fold compared to normal skin, and differentiated characteristics do not develop [12].

Since the discovery of the first microRNA (miRNA) gene, lin-4, in *Caenorhabditis elegans*, approximately 700 miRNA genes have been identified in humans [13]. MiRNAs play important roles in the regulation of development, proliferation, morphogenesis, apoptosis and differentiation. The role of miRNAs in skin development was demonstrated recently in a mouse model constructed with conditional knockout of Dicer in the skin [14], [15]. Yi et al. found more than 100 miRNAs in the skin and showed that epidermis and hair follicles differentially express discrete miRNA families [15]. Loss of keratinocyte-specific Dicer expression produces several distinct defects in the skin that affect both the epithelium and epithelial-mesenchymal signaling. These phenotypes include hyper proliferation in the absence of increased apoptosis [14], [15]. Hence, the importance of miRNAs in skin development is evident, although their precise functions in normal skin development and pathogenic conditions are unknown (reviewed in [16]).

The psoriatic phenotype is characterized by keratinocytes hyper proliferation and abnormal skin differentiation. Analysis of keratinocyte-derived miRNAs arrays revealed that hsa-miR-203 is up-regulated in psoriatic plaques [17]. Yet, many of the functions of miRNAs in the skin are largely unknown, and their involvement in the pathogenesis of skin disorders is even less understood. In this study we sought to identify miRNAs involved in the pathogenesis of psoriasis. We compared miRNA expression in normal skin to psoriatic involved and uninvolved skin and revealed 14 novel differentially expressed miRNAs. We further showed that IGF-1R, which has an important role in the psoriatic phenotype, is regulated by hsa-miR-99a.

## Results

### MiRNA expression analysis

To identify miRNAs involved in psoriasis, we compared miRNA expression in healthy skin to psoriatic involved an uninvolved skin using microarray technology. Full skin biopsies from three patients diagnosed with psoriasis were taken from psoriatic lesions as well as from uninvolved skin near the lesion. Normal skin biopsies were taken from three healthy volunteers. MiRNAs were extracted from the three biopsy types and samples from each type were pooled to reduce genetic variability.

**Figure 1:**
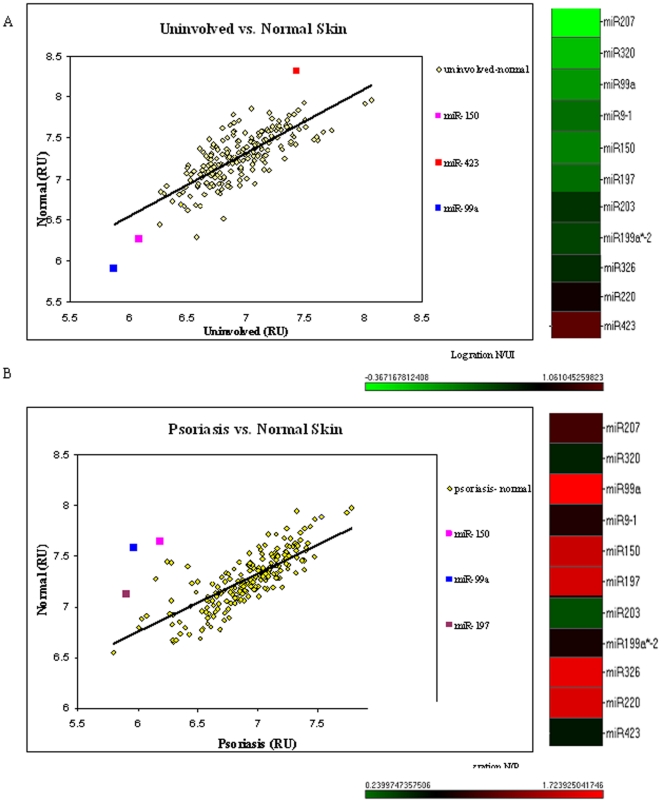
Profiling the expression level of miRNAs in psoriatic involved and uninvolved skin in comparison to normal skin. The expression of individual miRNAs in biopsies of normal skin, and psoriatic uninvolved and lesional skin was determined using miRNA expression array technology. The expression profile of normal skin was compared to uninvolved psoriatic skin (A) and psoriatic lesional skin (B). Abundance of an individual miRNA is shown by its relative position along the scaled y and x axes (arbitrary units). The colored boxes denote miRNAs which were further analyzed. See Table S1 for a detailed calculation of the heat-map.

The expression profiles of psoriatic uninvolved skin and lesion skin were compared to that of normal skin (Figure 1A and 1B, table S2 and table S1 respectively). As expected, the expression level of most of the miRNAs was the same. However, several miRNAs exhibited a significant difference in expression in both comparisons. The following differentially expressed miRNAs were specific to the normal versus psoriatic uninvolved skin comparison: hsa-miR-199a*2, hsa-mir-186, hsa-miR-22, hsa-miR-221 hsa-miR-220, hsa-miR-24-1, hsa-miR-296, hsa-miR-331, hsa-miR-324-5p, hsa-miR-329 hsa-miR-423 (hsa-miR-423 is marked as a red square in Fig. 1A). (Figure 1A and Table S3). The comparison between normal and psoriatic lesion skin revealed the following differentially expressed miRNAs: hsa-miR-149, hsa-miR-150, hsa-miR-210, hsa-miR-220, hsa-miR-326, has-miR-324-5p, hsa-miR-342, hsa-miR-326, hsa-miR-328, hsa-miR-345, hsa-miR-346, hsa-miR-197, (hsa-miR-197 is marked in brown in Fig 1B). (Figure 1B and Table S2). Hsa-miR-99a and hsa-miR-150 were found to be differentially expressed, more than a 2-fold difference, in both comparisons (Marked in blue and pink, respectively in Fig 1A and 1B).

In addition, the expression of hsa-miR-423 (Figure 1A) and hsa-miR-197 (Figure 1B) were dramatically changed, exhibiting more than a 2-fold difference in expression. Thus, these four miRNAs were chosen for further investigation. The array results are depicted in Table S1, S2, S3, S4.

### Validating the chip results

**Figure 2:**
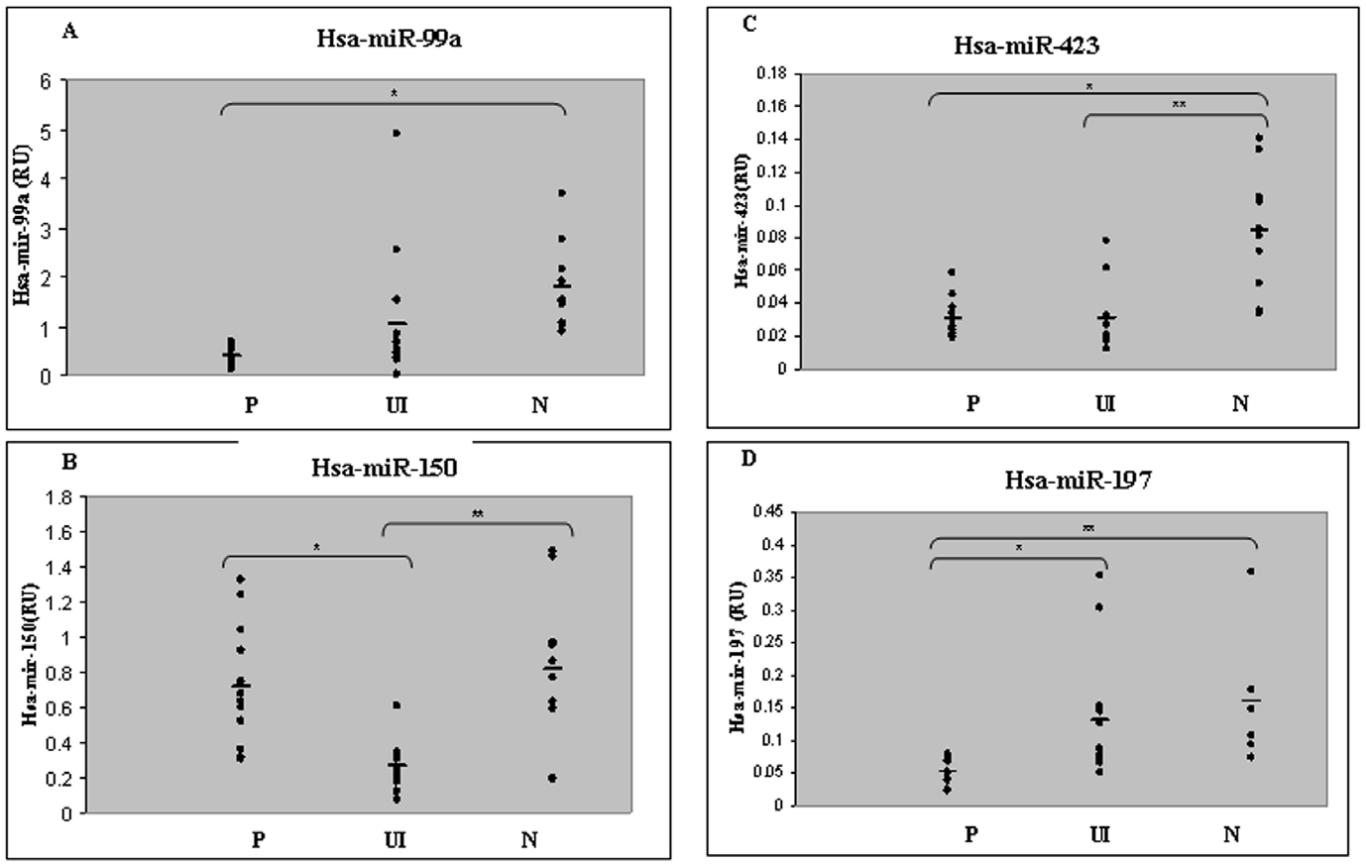
Quantification of miRNAs expression by qRT-PCR. The levels of hsa-miR-99a (A), hsa-miR-150 (B), hsa-miR-423 (C) and hsa-miR-197 (D) in normal skin (N, n = 10), psoriatic lesional skin (P, n = 16), and psoriatic uninvolved skin (UI, n = 16) were quantified by qPCR, and normalized to Rnu48. Each dot represents one sample. The average is denoted by the horizontal line. (A) Hsa-miR-99a (*p<0.0001 using un-paired t-test). (B) Hsa-miR-150 (*p<0.0007 using paired t-test, **p<0.0004 using un-paired t-test). (C) Hsa- miR-423 (*p<0.0001, **p<0.0003 using un-paired t-test). (D) Hsa-miR-197(**p<0.009 **p<0.0019 using un-paired t-test).

To verify the results obtained in the chip analysis, we assessed the expression of hsa-miR-99a, hsa-miR-150, hsa-miR-197 and hsa-miR-423 in normal and psoriatic uninvolved and lesion skin using qPCR. RNA was extracted from biopsies taken from 10 healthy volunteers and 16 psoriasis patients. As before, two biopsies were taken from each patient – one from the lesion and one from uninvolved skin. The expression of hsa-miR-99a was ∼4.5-fold lower in lesion skin and ∼2-fold lower in uninvolved skin as compared to normal skin (p<0.0001 P vs. N, using un-paired t-test, Figure 2A). Hsa-miR-150 was under expressed in uninvolved psoriatic skin, ∼3-fold less than in normal and psoriatic lesion skin (p<0.0007 in P vs. UI, using paired t-test, P<0.0004 in UI vs. N, using un-paired t-test, Figure 2B). Hsa-miR-423 was under expressed in uninvolved skin as well as in psoriatic lesion skin, ∼2.75 times less than in normal skin, (P<0.0001 in P vs. N , P<0.0003 in UI vs. N, using un-paired t-test, Figure 2C). Hsa-miR-197 was under expressed in psoriatic lesion skin, ∼3-fold less than in normal skin (P<0.0019 in P vs. N, using un-paired t-test). Its expression in uninvolved skin was similar to that in normal skin although the results were highly variable (Figure 2D). Not all miRNAs were down regulated in the psoriasis lesion; miR-203 was up regulated in psoriasis lesion samples as compared to normal skin samples (Supporting Information Figure S1) similar to previously published results [17]. Interestingly, we also detected a significant down regulation of miR-203 in the uninvolved psoriatic skin as compared to psoriatic lesion and normal skin (p<0.009 and 0.0001, respectively, Supporting Information Figure S1).

**Figure 3:**
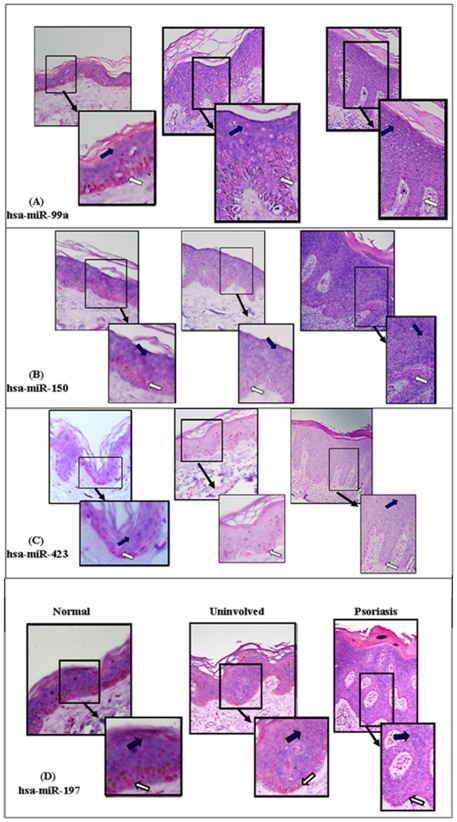
*in situ hybridization* of hsa-mir-99, hsa-mir-150, hsa-mir-423 and hsa-mir-197 in normal, uninvolved and psoriatic skin. Representative *in*-*situ* hybridization images showing distribution of hsa-miR-99a (A), hsa-miR-150 (B), hsa-miR-423 (C) and hsa-mir-197 (D) in psoriatic, uninvolved and normal skin. *In*-*situ* hybridizations were performed on paraffin-embedded sections of the three skin biopsy types. Each section was hybridized with an miRNA-specific LNA probe as indicated. Positive staining appears as a blue-purple color (examples are marked with blue arrows). Samples were co-stained with Nuclear Fast Red counter stain to visualize nuclei that are stained pink (examples are marked with white arrows). All images were captured at 200x magnification.

Next, we characterized the expression pattern of the four miRNAs in the epidermis. *In situ* hybridizations (ISH) were performed on biopsies of six psoriatic lesion skins, six uninvolved skins and five normal skins using specific LNA probes for hsa-miR-99a, hsa-miR-150, hsa-miR-423 and hsa-miR-197 (Figure 3). The specificity of the probes was confirmed by hybridization with an LNA scrambled-miRNA probe (data not shown). The ISH showed that the four miRNAs are present in keratinocytes and are differentially distributed throughout the epidermis. Hsa-miR-99a was found to be expressed in the upper part of the epidermis and excluded from the basal keratinocyte layer, in normal, uninvolved and psoriatic lesion skin (Figure 3A). Hsa-miR-150 was found to be expressed in the upper part of the epidermis and much less in the basal keratinocytes layer in normal skin. It was completely excluded from uninvolved skin, and appeared to be evenly distributed throughout the psoriatic epidermis (Figure 3B). Hsa-miR-423 appeared to be evenly distributed throughout the normal epidermis, and was hardly detected in the psoriatic lesion and uninvolved skin (Figure 3C). In contrast to miR-99a and miR-150, that were express gradually; higher in the upper epidermis and decreasing until complete absent from the basal layer, miR-197 was excluded from the basal layer of normal, uninvolved and psoriatic lesion skin and also was absent in the upper part of the epidermis in uninvolved and psoriatic lesion skin. At the rest of the epidermis miR-197 seemed to express equally (Figure 3D). The results of the ISH lack quantitative value due to variability in the intensity of staining among the samples. Nevertheless, the distribution of the four miRNAs was successfully demonstrated and the staining pattern was highly reproducible.

Our microarray and qPCR analysis revealed that hsa-miR-150 expression differs significantly between normal, psoriatic lesion and psoriatic uninvolved skin (Figures 1 and 2). Xiao et al. showed that miR-150 is selectively expressed in mature resting B and T cells, but not in their progenitors [18]. Our ISH analysis revealed that miR-150 is expressed in keratinocytes (Figure 3A). In addition we found that hsa-miR-150 is expressed both in HaCaT and PHK, reinforcing the fact that hsa-miR-150 is expressed in keratinocytes. However, miR-150 expression in keratinocytes is much lower than in B or T lymphocyte cell lines (data not shown).

### Reciprocal expression of IGF-1R and hsa-miR-99a

**Figure 4:**
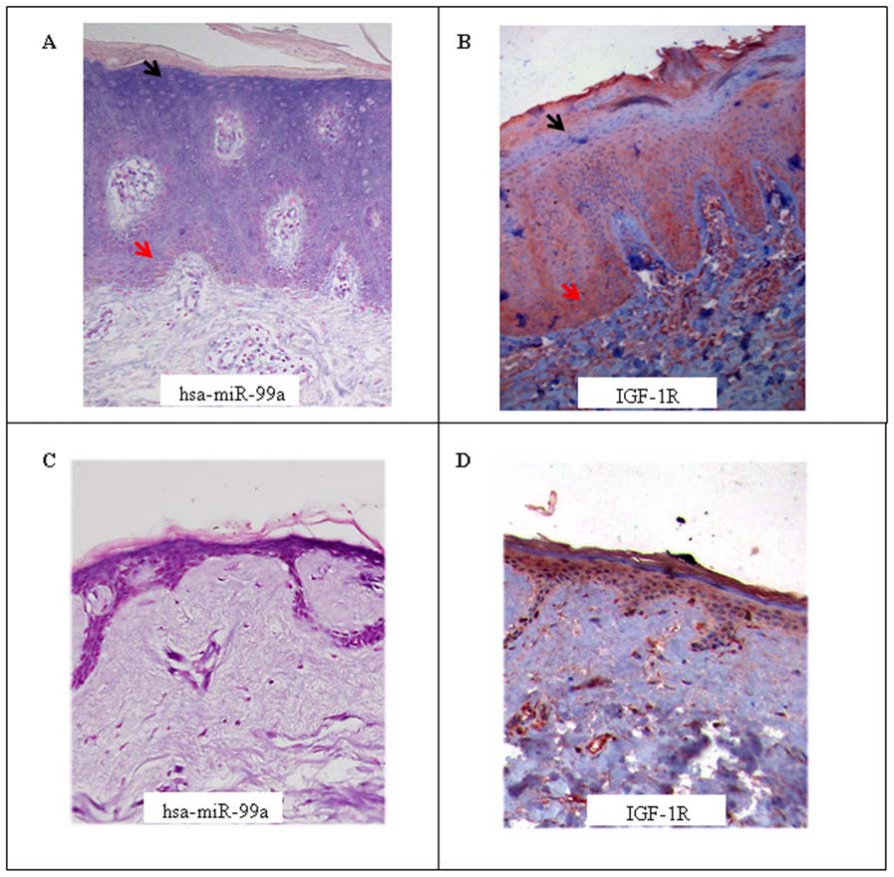
Reciprocal expression of IGF-1R and hsa-miR-99a. The distribution of hsa-miR-99a in psoriatic lesional skin (A) or normal skin (C) was assessed by *in*-*situ* hybridization. Paraffin-embedded sections of lesional skin biopsies were hybridized with an LNA probe specific to hsa-miR-99a. The cytoplasm of positive cells was stained blue (indicated by black arrows). Nuclei were stained with Nuclear Fast Red counter stain (indicated by red arrows). Magnification, 200x. (B) and (D) The same paraffin-embedded skin biopsy shown in (A and Crespectively;) was immunostained with anti-IGF-1R antibodies. Antibody binding was visualized with the substrate-chromogen AEC. IGF-1R positive cells were stained red (indicted by the red arrow). Nuclei were stained blue with hematoxylin (indicated by the black arrow). Magnification, 200x.

To understand the biological role of these differentially expressed miRNAs and their involvement in psoriasis, we sought to identify their possible targets. MiRNAs regulate gene expression mainly through interaction with the 3′UTR of a specific target mRNA. The binding specificity is directed by what is known as the miRNA ‘seed’ sequence: miRNA seeds are 7 to 8 nucleotides at the 5′ end that serve as the primary determinant of target specificity [19]. Based on this, several algorithms were developed to predict possible miRNA targets. We used these algorithms (miRanda, TargetScan, TargetRank) to predict possible targets for hsa-miR-99a. Between 40–950 predicted target mRNAs were found, one of which was IGF-1R, which was predicted by several algorithms. IGF-1R is known to be involved in the pathogenesis of psoriasis [20], [21]. IGF-1R was shown to be up-regulated in psoriasis, specifically in the proliferating basal layer of psoriatic lesions [20], [22]. Our ISH analysis revealed that hsa-miR-99a is not found in this layer (Figure 3A). We therefore decided to characterize the distribution of hsa-miR-99a and IGF-1R in the same sample to determine whether their expression pattern is reciprocal. ISH with specific LNA probes to hsa-mir-99a assay was performed on paraffin-embedded psoriatic lesion skin biopsies. Hsa-miR-99a was expressed in the upper part of the epidermis, most pronounced in the stratum spinosum and much less pronounced in the stratum basal layer (Figure 4A) also in normal skin (Figure 4C). The same biopsies were immunostained with anti-IGF-1R antibodies. IGF-1R was indeed up-regulated in the psoriatic lesions as compared to normal skin (Figure 4B and 4D) in agreement with previously published findings [20], [22]. Interestingly, IGF-1R was detected mainly in the basal layer of the epidermis, and was absent from the upper epidermal layer (Figure 4B). This reciprocal expression pattern suggests a possible relationship between hsa-miR-99a and IGF-1R.

**Figure 5:**
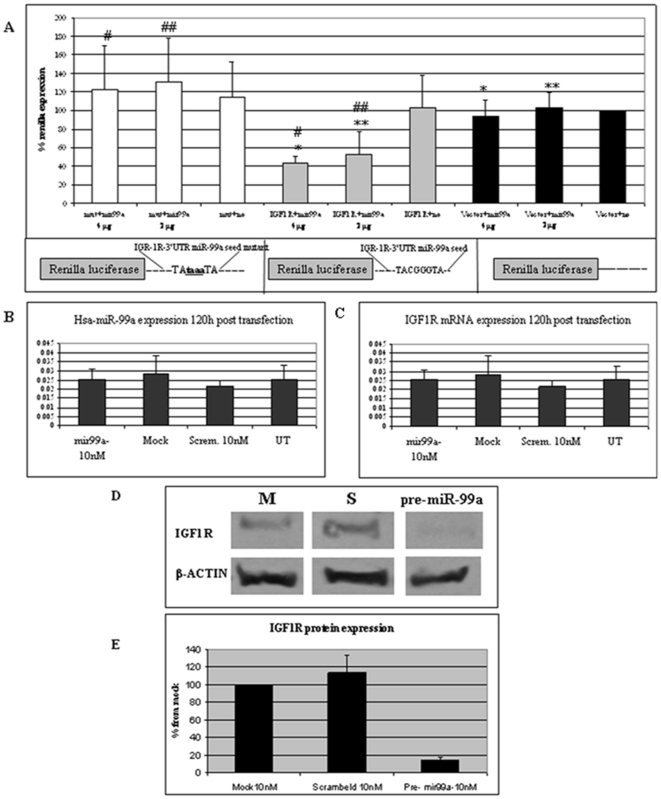
3′ UTR of IGF-1R is a target of hsa-miR-99a seed sequence. (A) 293T cells were transfected with psi-CHECK2 vectors encoding luciferase (right panel), luciferase fused to the IGF-1R 3′UTR (middle panel), or luciferase fused to a mutated IGF-1R 3′UTR (left panel) together with increasing amounts of an hsa-miR-99a expression plasmid as indicated. A scheme of the reporter vectors is shown under each panel. Luciferase activity was assessed 72 h later, and is depicted relative to that of the control cells transfected with psi-CHECK2 alone (given a value of 100%). Results represent the mean of triplicates. # p<0.0071, # # p<0.0047, * p<0.0075, ** p<0.0007. (B–C) PHK cells were transfected with 10 nM pre-miR-99a, a scrambled sequence, mock transfected or left untransfected. RNA was extracted 120 h later, and the level of hsa-miR-99a (B) and IGF-1R mRNA (C) was quantified by qPCR. Results represent the means of triplicates. Error bars, SD. (D) PHK cells were either mock-transfected or transfected with 10 nM pre-miR-99a, or a scrambled sequence. Cells were processed for Western blotting 120 h later to detect the level of IGF-1R protein. β-actin was used as the loading control. (E). The level of IGF-1R was quantified from the Western blot described in (D) by densitometry (EZQuant-Gel). Results represent the means of triplicate Western blots. Error bars, SD.

In order to determine whether IGF-1R is a direct target of hsa-miR-99a, we cloned a fragment of the IGF-1R 3′UTR mRNA downstream to the *Renilla* luciferase reporter gene [23]. Cells were either transfected with IGF-1R 3′UTR-luc alone, or together with increasing amounts of a hsa-miR-99a expression plasmid. The effect of miR-99a on the expression of the luciferase reporter containing the IGF-1R 3′UTR was assessed 72 h later with a standard luciferase reporter assay. Figure 5A (middle panel) shows that luciferase expression dropped to less than 50% in the presence of miR-99a, as compared to the control in the absence of miR-99a, or in the absence of the IGF-1R 3′UTR. These results suggest that hsa-miR-99a regulates the expression of IGF-1R through interaction with its 3′UTR. Next, we verified that the interaction between hsa-miR-99a and its predicted seed response sequence in the IGF-1R 3′UTR is responsible for down-regulating the expression of the reporter gene. We generated an IGF-1R-3′UTR-luc mutant in which four nucleotides in the seed response sequence were changed (TACGGGTA was changed to TA**TAAA**TA). The mutant was co-transfected with the miR-99a expression plasmid as before and luciferase activity was assessed. Figure 5A (left panel) clearly demonstrates that miR-99a has no effect on the mutated IGF-1R-3′UTR, proving that miR-99a seed sequence at the IGF-1R 3′UTR is essential for the regulation of IGF-1R by miR-99a. The effect of hsa-miR-99a on the regulation of IGF-1R was further examined by assessing IGF-1R mRNA and protein levels in primary human keratinocytes (PHK) transfected with RNA pre-miR-99a molecule. A scrambled sequence was used as the control. qPCR analysis showed that the level of IGF-1R mRNA in cells transfected with pre-miR-99a is similar to that in all the controls (untransfected, mock-transfected and scrambled-sequence-transfected cells; Figure 5B and C). In contrast, the level of IGF-1R protein was dramatically reduced in pre-miR-99a expressing cells, as determined by Western blot analysis (Figure 5D). Thus, IGF-1R is regulated at the post transcriptional level. Collectively, these results support our hypothesis that IGF-1R is a direct target of hsa-miR-99a.

### Hsa-miR-99a affects proliferation and differentiation of keratinocytes

**Figure 6:**
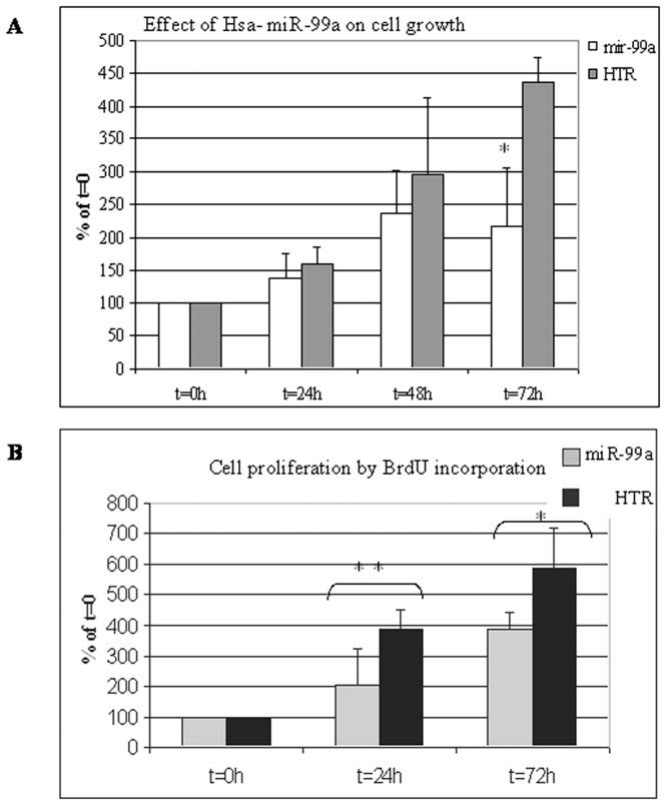
Hsa-miR-99a effect on keratinocytes cell growth. Proliferation of HaCaT cells stably expressing hsa-miR-99a or the HTR control RNA was assessed by MTT (A) and BrdU incorporation (B) assays. Proliferation was calculated as the percentage of the reading at seeding time (t = 0) given a value of 100%. Mean and standard deviation were calculated from 3 independent experiments. *P = 0.0037 in (A) and *P<0.0211**P<0.0420 in (B).

Thus far, we demonstrated the reciprocal expression pattern of hsa-miR-99a and IGF-1R in the epidermis (Figure 4), and provided evidence for the direct regulation of IGF-1R by hsa-miR-99a (Figure 5). The role of IGF-1R in proliferation of the skin is well established [24]. Our results suggest that miR-99a might be a key regulator of skin homeostasis. We first determined the effect of hsa-miR-99a on keratinocytes proliferation in the human keratinocyte cell line, HaCaT. Stable cell lines were established in HaCaT cells by transfections with an hsa-miR-99a expression plasmid (pMSCV-hsa-miR-99a) or a control plasmid expressing an unrelated small RNA molecule, HTR (pMSCV-HTR) followed by growth in selective medium [23]. The MTT assay was applied and viable cell count was evaluated over time. At 24 h after seeding, the growth rate of cells expressing hsa-mir-99a and HTR was similar. However, with time, the growth rate of the hsa-miR-99a expressing cells slowed down as compared to control cells. By 72 h, the hsa-miR-99a expressing cells had stopped proliferating, whereas the control cells continued to proliferate (Figure 6A). To confirm the effect of hsa-miR-99a on keratinocytes proliferation, we performed a BrdU incorporation assay in the two stable cell lines. The same trend demonstrated by the MTT assay was observed, but with different kinetics (Figure 6B). Thus, both proliferation assays demonstrate the inhibitory effect of hsa-miR-99a on keratinocyte proliferation.

**Figure 7:**
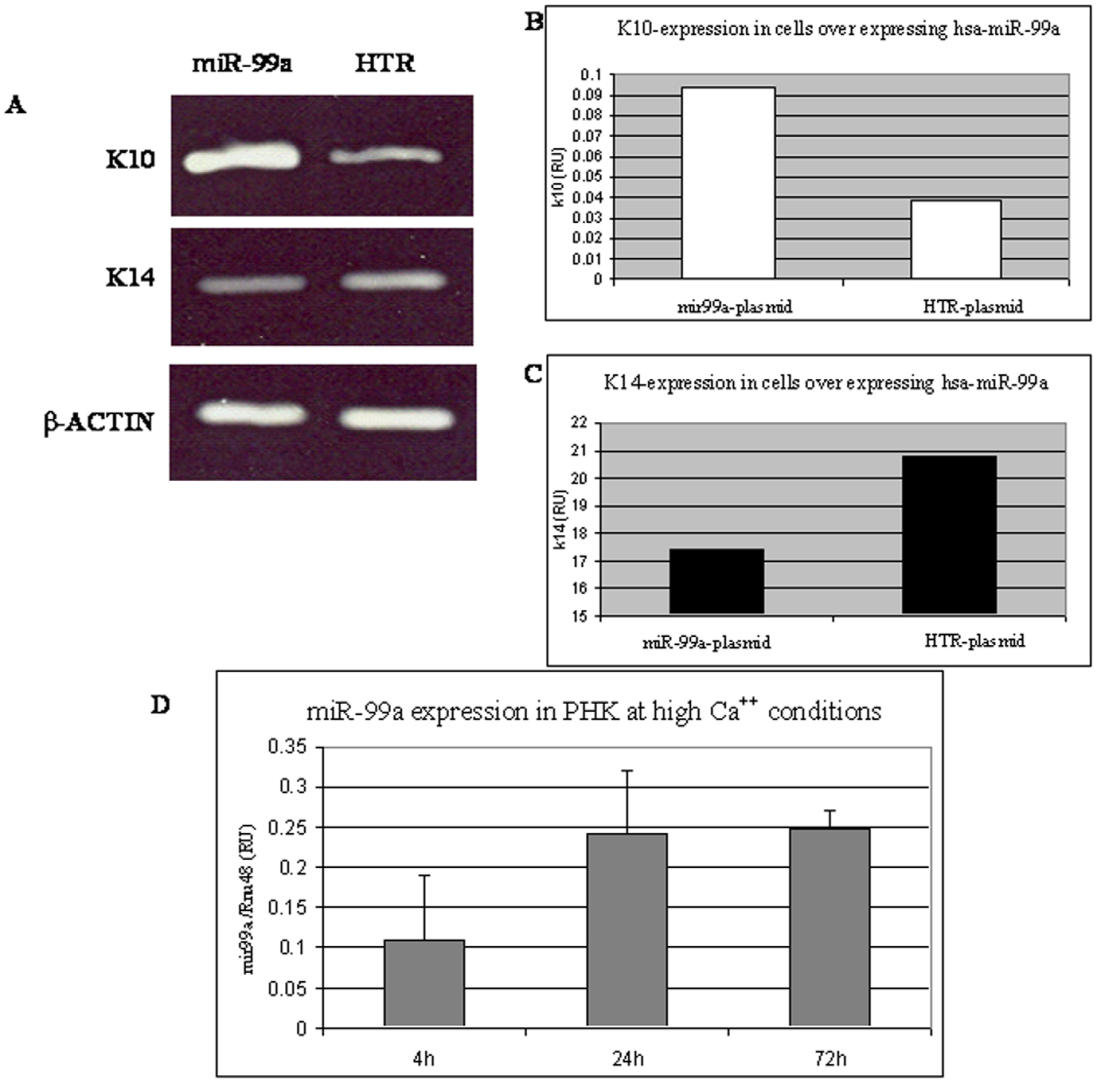
Hsa-miR-99a effect on differentiation of keratinocytes. (A–C) The levels of K10 and K14 differentiation markers were assessed in HaCaT cells stably expressing hsa-miR-99a or the HTR control RNA by RT-PCR (A) and qPCR (B–C) analysis. (D) PHK cells were seeded in high, 1.5 mM Ca^++^ medium. Cells were harvested at the indicated time points and the level of endogenous miR-99a was determined by qPCR.

Next, we determined the effect of hsa-miR-99a on keratinocyte differentiation. The expression of two keratinocyte differentiation related genes, Keratin14 (K14) and Keratin10 (K10), was evaluated in the two stable cell lines. K14 expression in normal skin is restricted to the basal layer, while K10 is expressed in more differentiated cells [25]. Total RNA was extracted from the two cell lines and RT-PCR was used to amplify K14 and K10 mRNA. Figure 7A shows that K10 expression is elevated and K14 is slightly decreased in the hsa-miR-99a expressing cells as compared to control cells. The same changes in expression were observed by qPCR analysis (Figure 7B and C). Furthermore, primary keratinocytes grown in high Ca^++^ medium, conditions known to induce keratinocyte differentiation [26], exhibited an increase in hsa-miR-99a expression over time, as determined by qPCR (Figure 7D).

We next determined the effect of hsa-miR-99a depletion on keratinocyte proliferation. A knockdown of miR-99a using antago-mir did not significantly change the growth rate or proliferation in PHK cells (data not shown). In addition, we used a sponge system to knockdown hsa-miR-99a in PHK cells [27], [28]. In order to verify that the miR-99a sponge is active, we co-transfected 293 cells with luciferase-IGF-1R-3′UTR and hsa-miR-99a expression plasmids together with variable amounts of the sponge expressing plasmid. As expected, luciferase expression was reduced to about 50% in cells transfected with the 3′UTR of IGF-1R and hsa-miR-99a as compared to control cells expressing hsa-miR-99a and the empty luciferase reporter plasmid. However, in the presence of the miR-99a sponge, the miR-99a-induced repression was released; the luciferase level in cells transfected with 4 µg of sponge plasmid was almost completely restored to that of the control (Supporting Information Figure S2). We generated cell lines of HaCaT cells stably expressing the miR-99a sponge or the empty vector control and assessed their proliferation rate using the MTT assay. No significant difference in proliferation rate was observed between the miR-99a sponge expressing and control cell lines (Supporting Information Figure S2). However, a minor but not significant enhancement of IGF-1R protein level was detected in the miR-99a sponge expressing cell line as compared to the control cell line (Supporting Information Figure S2), in agreement with our results demonstrating that IGF-1R is a target of miR-99a (Figure 5). We also determined whether the miR-99a sponge has an effect on keratinocyte differentiation. As shown in Supporting Information Figure S2, there is no change in expression of keratin-10 or keratin-14.

**Figure 8:**
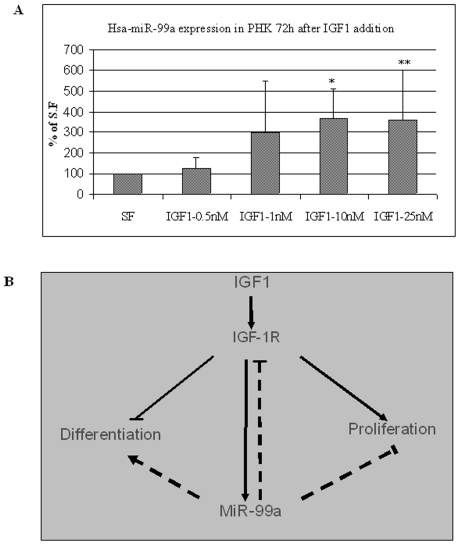
hsa-miR-99a expression in primary human keratinocytes 72 h after IGF1 addition. (A) Serum-starved PHK cells were treated with increasing concentrations of IGF-1 as indicated. The cells were harvested 72 h later and the level of endogenous miR-99a was determined by qPCR (*p = 0.0027 **p = 0.04510). (B) Proposed model describing the relationship between IGF-1R and miR-99a. IGF1 activation of IGF-1R inhibits differentiation and promotes proliferation of keratinocytes [24]. IGF-1R signaling enhances the expression of miR-99a that in turn inhibits IGF-1R expression. This negative feedback loop maintains the balance between proliferation and differentiation in the skin.

These results indicate that the over-expression of hsa-miR-99a slows keratinocyte growth rate and directs them towards differentiation. As shown in Figure 5, miR-99a regulates the expression of IGF-1R. IGF-1R was shown to have a major role in keratinocyte differentiation [24]. Considering these two facts, we thought that the expression of miR-99a and IGF-1R might be correlated. To further investigate the relationship between miR-99a and IGF-1R, we assessed miR-99a expression in serum-starved PHK cells treated with increasing concentrations of IGF1. Total RNA was extracted 72 h after IGF1 addition, and the miR-99a level was determined by qPCR. The expression of miR-99a was significantly increased in cells treated with 1–25 nM of IGF1 as compared to untreated cells (Figure 8A). Collectively, our results suggest that miR-99a and IGF-1R are co-regulated, functioning together to maintain the balance between keratinocyte proliferation and differentiation.

## Discussion

MiRNAs are major regulatory molecules that govern many essential cellular functions, including proliferation, differentiation, cell cycle control and apoptosis. The significance of miRNAs in skin development was demonstrated by Yi et al., who showed that skin formation is impaired in keratinocyte-specific Dicer knockout mice [14], [15]. A recent comparison of miRNA expression profiles of psoriatic and normal or atopic dermatitis skins revealed several leukocyte-derived miRNAs and one keratinocyte-derived miRNA, miR-203, which were up-regulated in psoriatic skin [17]. The functions of miR-203 seem to be related to keratinocyte stem cell differentiation [29]. Furthermore, the transcription factor p63, which plays an essential role in epidermis formation, is a target of miR-203 [29]. Our qPCR analysis of miR-203 expression demonstrated that it was up regulated in psoriatic skin compared to normal skin in agreement with previously published findings [17]. Unpredictably, hsa-miR-203 was significantly down regulated in uninvolved psoriatic skin compared to normal as well as psoriatic skin (Supporting Information Figure S1). This result implies that down regulation of miR-203 in uninvolved psoriatic skin may predispose psoriatic uninvolved keratinocytes to hyper proliferation and should be investigated in further studies.

Our comparison of miRNA expression profiles in normal, psoriatic lesion and uninvolved skin revealed several novel candidate miRNAs that might be involved in the pathogenesis of psoriasis (Figure 1). We focused our investigation on hsa-miR-99a, hsa-miR-150, hsa-miR-197 and hsa-miR-423, as they exhibited a significant difference in expression (more than 2-fold) (Figure 1 and Supporting Information Table S1). We verified the expression of these four miRNAs as well as miR-203 in skin biopsy types using qPCR, confirming their differential expression in normal and psoriatic skin (Figure 2 and Supporting Information Figure S1). Interestingly, miR-203 and miR-150 were down-regulated in uninvolved skin as compared to normal skin or lesional skin (Figure 2B and Supporting Information Figure S1), suggesting that in uninvolved skin of psoriasis patients down regulation of these two miRNAs predisposes them to the psoriatic phenotype. The expression of hsa-miR-99a, hsa-miR-423 and hsa-miR-197 in the psoriasis lesion were down-regulated compared to normal skin. However, their average expression in the uninvolved skin of psoriasis patients differed; average expression of miR-99a was somewhere between normal and lesion expression levels (Figure 2A), expression of miR-423 was down regulated in uninvolved skin as in the lesion skin in comparison to normal skin (Figure 2C) and miR-197 expression in uninvolved skin was similar to that in normal skin (Figure 2D). This might suggest a biochemical hierarchy in the development of the psoriatic lesion.

We characterized the distribution of the four miRNAs in the epidermis by performing ISH on skin biopsies. This analysis revealed that the four miRNAs are expressed in keratinocytes. In addition, all four miRNAs were either absent or weakly expressed in the basal keratinocyte layer, whereas only hsa-miR-99a, hsa-miR-150 and hsa-miR-423 were present in the upper part of the epidermis (Figure 3). This distribution suggests that these miRNAs have the potential to regulate keratinocyte proliferation and differentiation.

Hsa-miR-150 was shown to be involved in immune cell function. It is selectively expressed in mature resting B and T cells, controlling B-cell differentiation by targeting the transcription factor c-Myb [18]. The involvement of the immune system in psoriasis is well established [10], [30], [31], [32], so it is possible that hsa-miR-150 targets immune system regulators in the skin. A possible target of hsa-mir-150 is IL-7 that is expressed in keratinocytes [33] and is up-regulated in psoriatic lesions [34]. Another possible target is IRAK2 encoding the interleukin-1 receptor-associated kinase 2 that was reported to participate in the IL1-induced up-regulation of NF-kappaB, a pathway known to be involved in the pathogenesis of psoriasis [35]. In addition to a possible role in immune system regulation, hsa-miR-150 might regulate keratinocyte proliferation. Indeed, Wu et al. found that miR-150 promotes gastric cancer proliferation by negatively regulating the pro-apoptotic gene EGR2 [36]. Further investigation is required to identify the targets of hsa-miR-150 in keratinocytes.

We focused on identifying the targets of hsa-miR-99a, the miRNA exhibiting the strongest down-regulation in psoriatic skin (Figure 2A). We used bioinformatics tools to predict possible targets of hsa-miR-99a, and decided to focus on one predicted target, IGF-1R, that is known to be involved in the pathogenesis of psoriasis [20], [21]. ISH revealed that both hsa-mir-99a and IGF-1R are expressed in the psoriatic lesion. However, the distribution of their expression is reciprocal, with a high expression of IGF-1R in the basal epidermis layers, from which hsa-miR-99a is absent, and a high expression of hsa-miR-99a in the upper epidermis, from which IGF-1R is absent (Figure 4). Furthermore, we used a standard luciferase reporter assay to demonstrate that the 3′UTR of IGF-1R is regulated by hsa-miR-99a and that this regulation is dependent on the interaction between the hsa-miR-99a seed and the IGF-1R mRNA 3′UTR (Figure 5A). Moreover, we showed that over expression of hsa-miR-99a reduces endogenous IGF-1R protein levels without affecting its transcript levels, demonstrating post-transcriptional regulation of IGF-1R (Figure 5C and D). Thus, we provide strong evidence for the direct regulation of IGF-1R by hsa-miR-99a.

MiRNAs are highly expressed in embryonic tissues [37] and are known to have a key role in differentiation and development. Two main characteristics of psoriasis are keratinocyte hyper proliferation and abnormal differentiation. IGF-1R plays an important role in keratinocyte differentiation by actively inhibiting the differentiation process [24]. Ablation of IGF-1R expression eliminates this inhibition, resulting in facilitated differentiation [24]. Our results suggest that hsa-miR-99a contributes to the homeostasis of IGF-1R expression. We hypothesize that the elevation of IGF-1R in the psoriatic lesion drives the tissue towards proliferation. One of the possible physiological responses to such a situation is up-regulation of hsa-miR-99a expression which may slow keratinocyte proliferation and induce their differentiation. Indeed, HaCaT cells transfected with hsa-miR-99a expression plasmid exhibited a decreased proliferation rate and an increase in expression of a late differentiation marker as compared to control cells (Figures 6 and 7A–C). Moreover, miR-99a expression was up regulated in differentiating PHK cells grown in high calcium medium (Figure 7D). We also examined the effect of down regulating hsa-miR-99a in HaCaT cells. Using a sponge system, we demonstrated that IGF-1R expression is up-regulated in hsa-miR-99a-depeleted cells (Supporting Information Figure S2), however the proliferation rate was unchanged (Supporting Information Figure S2). It is possible that the combined effect of several regulators is required to achieve the hyper proliferation phenotype. Lastly, the relationship between hsa-miR-99a and IGF-1R was further demonstrated by an increase in endogenous hsa-miR-99a expression in PHK cells treated with IGF-1 (Figure 8A). We propose a model to describe how hsa-miR-99a and IGF-1R work together to achieve a balance between proliferation and differentiation in the skin (Figure 8B). Activation of IGF-1R in skin enhances proliferation and inhibits differentiation. Over time, IGF-1R signaling up-regulates the expression of miR-99a that in turn down-regulates the expression of IGF-1R, driving the cells toward differentiation. Is the entire effect of miR-99a on keratinocyte differentiation mediated through its effect on IGF-1R? It is possible; however, it is not probable, since miRNAs usually have more than one target. Our model fits well with the work of Yi et al in which miR-203 was given a comparable role in restricting proliferation and inducing differentiation of keratinocytes [38]. Based on our results and previous studies, we suggest that keratinocytes in the psoriatic lesion are prompted to proliferation, possibly by mediators produced by immune system cells. These mediators may either directly or through the involvement of stromal cells, enhance over proliferation of keratinocytes in the basal layer. During the passage of these cells from the basal layer into the stratum spinosum, differentiation signals are activated; our study describes one such signal, involving hsa-miR-99a-mediated suppression of IGF-1R expression.

In addition we found that some of the miRNAs we analyzed are expressed differentially in the uninvolved skin compared to normal skin. These results prove that the uninvolved skin of psoriasis patients differs from normal skin. This fact suggests that the regulation of miRNA expression plays a major role in the pathogenesis of psoriasis. Are the psoriatic uninvolved keratinocytes more susceptible to inflammatory mediators? It is interesting to further investigate if these differences can be used as diagnostic markers or as therapeutic agents.

This study raises additional questions: Is the activation of these miRNAs specific to the psoriasis lesion? The process by which proliferating keratinocytes eventually differentiate is common to several additional inflammatory skin disorders and wound healing. Are the same miRNAs involved in all these biological processes, or do specific miRNAs act in specific processes? The answers to these questions will shed light on our understanding of the differences between these disorders.

## Materials and Methods

### Patients

All skin donors were of Caucasian origin aged 18 to 85 years (Table S5). All patients were clinically diagnosed with Psoriasis Vulgaris, and did not receive systemic immunosuppressive treatment, phototherapy (Psoralen and UVA (PUVA)/solarium/UVB), or topical therapy for at least 3 weeks prior to skin biopsy (for further details see, Table S5). Three mm punches were taken mostly from upper and lower limbs. The uninvolved samples were taken from the same area, about 5 cm away from the lesion biopsy. Normal skin biopsies were taken from full thickness skin remaining after plastic surgery. All the biopsies were evaluated by a dermatopathologist for histological diagnosis. Half of each biopsy was snap-frozen and the other half was embedded in paraffin. The study was approved by both the Tel Aviv University and Sheba Medical Center Helsinki Ethics Committees. All participants provided written informed consent.

### MiRNA array

Total RNA including miRNAs from normal skin (n = 3), psoriatic lesion (n = 3), or psoriatic patient uninvolved skin (n = 3), was isolated using Ambion mirVana™ miRNA Isolation Kit. Total RNA (2 µg) from each sample was labeled with the mirVana miRNA Labeling Kit (Applied Biosystems/AmbionUSA). The fluorescently-labeled RNA samples were hybridized to an expression array as previously described [39]. The array was scanned and analyzed using Genepix pro 4000b Axon and JMP statistic software.

### Cells cultures

293T and HaCaT cells were grown in DMEM and MEM medium, respectively, supplemented with glutamine, antibiotics and 10% fetal bovine serum (Biological Industries, Israel) in a 5–8% CO_2_ incubator at 37°C.

PHK cells were grown in an 8% CO_2_ incubator at 37°C, in high Ca^++^ (1.5 mM) medium: DMEM / HAM (Biological Industries, Israel) /10%FBS (Gibco). Keratinocytes were harvested from mashed skin (left over from plastic surgery) and grown on a feeder layer of 5000 rad γ irradiated 3T3 mice fibroblast cells.[40].

### Cell growth and proliferation assays

Cell growth was assessed by seeding 3000 HaCaT cells per well in 96-well plates. Viable cell counts were monitored from seeding time (t = 0) to 72 h. Cell counts were determined using the MTT (3-[4, 5-dimethylthiazol-2-yl]-2, 5-diphenyl tetrazolium bromide)-based Cell Growth Determination Kit TOX-1 (Sigma-Aldrich, Israel Ltd. Rehovot 76100 ISRAEL) and the BrdU colorimetric kit (cat. 11 647 229001Roche), according to the manufacturer's instructions Each experiment was performed in triplicate.

### Quantitative real time PCR (qPCR)

Total RNA of skin biopsies and cells was extracted using Ambion *mirVana*™ miRNA Isolation Kit. Quantification of miRNAs by *TaqManH* Real-Time PCR was performed on 10 ng RNA using the ABI 7900HT thermocycler (Applied Biosystems) for 40 cycles. Target miR/ gene expression was normalized between different samples based on the values of RNU48/Rplpo expression respectively.

### *In situ* hybridization

*In situ* transcriptional levels of the different miRNAs were evaluated on paraffin-embedded skin biopsy specimens from 3 healthy skins and 5 psoriasis patients volunteers. Formalin-fixed tissues were dehydrated, embedded in paraffin and sectioned at 4 µm. A section of a healthy volunteer skin biopsy and 2 sections of involved and uninvolved skin from a psoriatic patient were placed on each slide. The slides were warmed up to 60°C for 1 h, de-waxed in xylene and rehydrated. Sections were hybridized overnight with digoxygenin-labeled miRCURY LNA probes (Exiqon, Denmark), and incubated with anti-digoxygenin antibody conjugated with alkaline phosphatase for 1 h. The color reaction was performed at 1–48 h. Counter staining was done by Nuclear Fast Red counter stain. The stained sections were reviewed with a light microscope and analyzed by a dermato pathologist who was blind to the tissue origin.

### IGF-1R immunostaining

The formalin-fixed tissues were treated in the same manner as the ISH slides. Antigen retrieval was performed using a pressure cooker (Milestone, Microwave Laboratory Systems) at 120°C for 5 min in 0.1 M citrate buffer pH 6. The slides were blocked in 3% H2O2/MeOH and then incubated with primary antibody to IGF-1R (Abcam, ab54274, 1:750). Detection was performed with the Histostain SP Broad Spectrum kit (Zymed Laboratories, Inc. USA). The antibody binding was visualized with the substrate-chromogen AEC, counterstained with hematoxylin.

### Cloning and plasmids

The plasmids pMSCV-miR-99a and pMSCV-HTR were kindly provided by Agami, R.[23] ****.

A genomic fragment containing 1070 bp of the 3′UTR of IGF-1R mRNA (NCBI Reference Sequence: NM_000875.3) from position 9251 to 10320 was amplified by PCR using the following primers:

1-5′-ACAGGTCAGAGGGTTTC-3′; 2- 5′-CAGTCAAGTCTTCCCATGT-3′.

PCR fragments were cloned into pCRII-TOPO plasmid (Invitrogen,USA). The XhoI fill-in KpnI fragment was cloned into psiCHECK-2 plasmid (Promega,USA) digested with XhoI and PmeI.

An IGF-1R 3′UTR mutant for the hsa-mir-99a seed sequence was created using the Megaprimer Mutagenesis assay [41], [42] using primer 1 and primer 3: 5′-TAGAT**TATAAATA**GTCAGTT-3′.

The hsa-miR-99a over expression oligo, Pre-miR miRNA precursor, was purchased from Ambion- Applied Biosystems. The miR-99a sponge expression plasmid was generated as follows: two complementary oligo nucleotides of ∼120 bases were synthesized and annealed generating double stranded DNA containing four hsa-mir-99a “sponge” sequences with XhoI and XmaI restriction sites on either end. The double stranded DNA was cloned into XhoI and XmaI sites of pTurbofp635-c plasmid (Evroege, Russia). DNA synthesis and the cloning was performed by GenScript USA.

### Determination of mRNA levels by RT-PCR

Total RNA was extracted using *mirVana*™ isolation kit (Applied Biosystems/Ambion, USA). RT-PCR was performed using the Verso thermo-scientific kit using the following primers:

krt14: 5′-AGCAGCAGAACCAGGAGTAACAAG-3′

5′-GGCGTAGGTGGCGATCT-3′

krt10: 5′- GCTGACATCAACGGCCT-3′

5′- AGCATTCATTTCCACATTCAC-3′

β-actin: 5′- CCTGGCACCCAGCACAAT -3′

5′- GCCGATCCACACGGAGTACT -3′

### Transfections

293T cells were seeded at 0.5–1×10^5^ cells per well and transfected by calcium phosphate in HEPES buffer method, as described [43].

HaCaT cells were transfected using Lipofectamine™ 2000 Reagent (Invitrogen,USA).

### Luciferase Assay

Luciferase assay was performed 72 h post transfection using the The Dual-Luciferase® Reporter (DLR) Assay System (Promega,USA).

### Determination of protein expression level by Western blotting (WB)

WB was performed as described previously [44], using primary specific antibody (IGF-1Rβ c20 CS-713 Santa Cruz Biotechnology, USA)/ (Beta Actin AC-15 (Abcam UK).

## Supporting Information

Figure S1 The level of hsa-miR-203 in normal skin (N, n = 10), psoriatic lesional skin (P, n = 16), or psoriatic uninvolved skin (UI, n = 16) was determined by qPCR and normalized to Rnu48. Y bars are arbitrary units that define fold change. Each dot represents one sample. Average is denoted by the horizontal line. *P<0.009,**p<0.0001.
(DOC)

Figure S2 A) 293T cells were co-transfected with 10 ng psiCheck vector, or psiCheck-IGF-1R-3′UTR plasmid, together with 2 µg of hsa-miR-99a expressing plasmid. In addition cells were transfected with 0 µg, 2 µg, or 4 µg of miR-99a sponge expressing plasmid. 48 hours post transfection cells were harvested and subject to Dual-Luciferase Reporter assay. Each experiment was done in triplicates. The average of 3 wells transfected with vector lacking the IGF-1R 3′UTR and without miR-99a sponge expressing plasmid (vector+0) was valued as 100%. The average is of 4 independent experiments. Paired *t* test results; # p<0.0044, * p<0.0052. B) HaCaT cells were stably transfected with plasmid expressing miR-99a sponge, or with the vector plasmid. After, almost two months of G418 selection, the same amount of cells from each plate were harvested and subjected to Western blot analysis, using either IGF1R or actin antibodies, the graph is average of three experiments; densitometry and calculation were done with ImageJ. C) MTT assay of HaCaT cells expressing the miR-99a sponge plasmid (sponge) or the empty vector (vector). 4000 cells were seeded in each well of 96 well plates. 4 h after seeding MTT was added and counted as time 0. The graph represents an average of three experiments, in each experiment every time point is an average of three wells. D) Total RNA was extracted form vector expressing or miR-99a sponge expressing cells, and was subject to RT-PCR using specific primers as indicated, the graph is average of three experiments; densitometry and calculation were done with ImageJ.
(DOC)

Table S1 Summary of miRNAs that were change in each of the comparisons also marked whether these miRNAs were up regulated, or down regulated, or unchanged. In each miRNA the fold change is noted, marked in yellow these miRNAs that were chosen for further analysis. In the comparison of normal to psoriatic skin also added miRNAs that were shown to change by Sonkoly et. al.[2]. Among these miR-133a was marked as miRNA that did change in there work by 1.76 fold, and in our screening miR-133a did change by 1.8 fold (marked in blue), however, we defined as change two fold or more.
(DOC)

Table S2 **Comparison of normal versus psoriatic lesional skin.** Total RNA from normal skin (n = 3), psoriatic lesional (n = 3), was isolated using Ambion mirVana™ miRNA Isolation Kit. Two µg of total RNA from each sample were labeled with the mirVana miRNA Labeling Kit (Applied Biosystems/Ambion Austin, TX 78744-1832, USA). The normal was labeled red and the psoriasis green. The fluorescently-labeled RNA samples were hybridized to an expression array as previously described [1]. The array was scanned and analyzed using Genepix pro 4000 b Axon and JMP statistic software. After background-subtracted, normalized spot intensities presented as log_e_ of the scanned value, ratio calculated of N to P for individual spot is note (Each miRNA is represented on the CHIP either 4 or 8 times; the value is an average of all spots of the same miRNA). MiRNAs that were found to change more than two fold are highlighted as well as miRNAs that were further analysis.
(DOC)

Table S3 **Comparison of normal versus uninvolved psoriatic skin.** Total RNA from normal skin (n = 3), or psoriatic uninvolved skin (n = 3) was isolated using Ambion mirVana™ miRNA Isolation Kit. Two µg of total RNA from each sample were labeled with the mirVana miRNA Labeling Kit (Applied Biosystems/Ambion Austin, TX 78744-1832, USA). The normal was labeled green and uninvolved red. The fluorescently-labeled RNA samples were hybridized to an expression array as previously described [1]. The array was scanned and analyzed using Genepix pro 4000 b Axon and JMP statistic software. After background-subtracted, normalized spot intensities presented as log_e_ of the scanned value, ratio calculated of N to UI or for individual spot is note (Each miRNA is represented on the CHIP either 4 or 8 times; the value is an average of all spots of the same miRNA). MiRNAs that were found to change more than two fold are highlighted as well as miRNAs that were further analysis.
(DOC)

Table S4 **Comparison of psoriatic versus uninvolved psoriatic skin.** Calculation were taken from tables S2 and table S3 After background-subtracted, normalized spot intensities presented as log_e_ of the scanned value, ratio calculated of UI to P for individual spot is note (Each miRNA is represented on the CHIP either 4 or 8 times; the value is an average of all spots of the same miRNA). MiRNAs that were found to change more than two fold are highlighted as well as miRNAs that were further analysis.
(DOC)

Table S5 **Patient characteristics.**
(DOC)
