# Supplementary material for: MiRNA Expression in Psoriatic Skin: Reciprocal Regulation of hsa-miR-99a and IGF-1R
Source: PLoS One. 2011 Jun 7;6(6):e20916. doi: 10.1371/journal.pone.0020916 (PMC3110257; doi:10.1371/journal.pone.0020916)
Supplement: Figure S1 — The level of hsa-miR-203 in normal skin (N, n = 10), psoriatic lesional skin (P, n = 16), or psoriatic uninvolved skin (UI, n = 16) was determined by qPCR and normalized to Rnu48. Y bars are arbitrary units that define fold change. Each dot represents one sample. Average is denoted by the horizontal line. *P<0.009,**p<0.0001. (DOC) [file pone.0020916.s001.doc]

**Figure S1**: The level of hsa-miR-203 in normal skin (N, n=10), psoriatic lesional skin (P, n=16), or psoriatic uninvolved skin (UI, n=16) was determined by qPCR and normalized to Rnu48. Y bars are arbitrary units that define fold change. Each dot represents one sample. Average is denoted by the horizontal line. *P<0.009,**p<0.0001.
