# Supplementary material for: MiRNA Expression in Psoriatic Skin: Reciprocal Regulation of hsa-miR-99a and IGF-1R
Source: PLoS One. 2011 Jun 7;6(6):e20916. doi: 10.1371/journal.pone.0020916 (PMC3110257; doi:10.1371/journal.pone.0020916)
Supplement: Figure S2 — A) 293T cells were co-transfected with 10 ng psiCheck vector, or psiCheck-IGF-1R-3′UTR plasmid, together with 2 µg of hsa-miR-99a expressing plasmid. In addition cells were transfected with 0 µg, 2 µg, or 4 µg of miR-99a sponge expressing plasmid. 48 hours post transfection cells were harvested and subject to Dual-Luciferase Reporter assay. Each experiment was done in triplicates. The average of 3 wells transfected with vector lacking the IGF-1R 3′UTR and without miR-99a sponge expressing plasmid (vector+0) was valued as 100%. The average is of 4 independent experiments. Paired t test results; # p<0.0044, * p<0.0052. B) HaCaT cells were stably transfected with plasmid expressing miR-99a sponge, or with the vector plasmid. After, almost two months of G418 selection, the same amount of cells from each plate were harvested and subjected to Western blot analysis, using either IGF1R or actin antibodies, the graph is average of three experiments; densitometry and calculation were done with ImageJ. C) MTT assay of HaCaT cells expressing the miR-99a sponge plasmid (sponge) or the empty vector (vector). 4000 cells were seeded in each well of 96 well plates. 4 h after seeding MTT was added and counted as time 0. The graph represents an average of three experiments, in each experiment every time point is an average of three wells. D) Total RNA was extracted form vector expressing or miR-99a sponge expressing cells, and was subject to RT-PCR using specific primers as indicated, the graph is average of three experiments; densitometry and calculation were done with ImageJ. (DOC) [file pone.0020916.s002.doc]

0

2μg

4μg

**IGF-1R-3'UTR +2μg of miR-99a**

**Vector +2μg of miR-99a**

#

*

*

A

#

C

D

B

**Figure S2:** A) 293T cells were co-transfected with 10ng psiCheck vector, or psiCheck-IGF-1R-3'UTR plasmid, together with 2µg of hsa-miR-99a expressing plasmid. In addition cells were transfected with 0µg, 2µg, or 4µg of miR-99a sponge expressing plasmid. 48 hours post transfection cells were harvested and subject to Dual-Luciferase Reporter assay. Each experiment was done in triplicates. The average of 3 wells transfected with vector lacking the IGF-1R 3'UTR and without miR-99a sponge expressing plasmid (vector+0) was valued as 100%. The average is of 4 independent experiments.

Paired *t* test results; # p< 0.0044, * p<0.0052.

B) HaCaT cells were stably transfected with plasmid expressing miR-99a sponge, or with the vector plasmid. After, almost two months of G418 selection, the same amount of cells from each plate were harvested and subjected to Western blot analysis, using either IGF1R or actin antibodies, the graph is average of three experiments; densitometry and calculation were done with ImageJ.

C) MTT assay of HaCaT cells expressing the miR-99a sponge plasmid (sponge) or the empty vector (vector). 4000 cells were seeded in each well of 96 well plates. 4 h after seeding MTT was added and counted as time 0. The graph represents an average of three experiments, in each experiment every time point is an average of three wells.

D) Total RNA was extracted form vector expressing or miR-99a sponge expressing cells, and was subject to RT-PCR using specific primers as indicated, the graph is average of three experiments; densitometry and calculation were done with ImageJ.
