# Supplementary material for: MiRNA Expression in Psoriatic Skin: Reciprocal Regulation of hsa-miR-99a and IGF-1R
Source: PLoS One. 2011 Jun 7;6(6):e20916. doi: 10.1371/journal.pone.0020916 (PMC3110257; doi:10.1371/journal.pone.0020916)
Supplement: Table S1 — Summary of miRNAs that were change in each of the comparisons also marked whether these miRNAs were up regulated, or down regulated, or unchanged. In each miRNA the fold change is noted, marked in yellow these miRNAs that were chosen for further analysis. In the comparison of normal to psoriatic skin also added miRNAs that were shown to change by Sonkoly et. al.[2]. Among these miR-133a was marked as miRNA that did change in there work by 1.76 fold, and in our screening miR-133a did change by 1.8 fold (marked in blue), however, we defined as change two fold or more. (DOC) [file pone.0020916.s003.doc]

**Table S1:** summary of miRNAs that were change in each of the comparisons

| Comparison of normal to psoriasis | | | | | |
| --- | --- | --- | --- | --- | --- |
| Fold change | Unchanged miRNAs | Fold change | miRNAs down  regulated in normal | Fold change | miRNAs up  regulated in normal |
| 1.42 | Mir-423 |  |  | 3.3 | Mir-149 |
| 1.5 | Mir-203 |  |  | 4.27 | Mir-150 |
| 1.27 | Mir-146 |  |  | 2.35 | Mir-187 |
| 1.06 | Mir-31 |  |  | 3.35 | Mir-197 |
| 1.27 | Mir-125b-1 |  |  | 3.17 | Mir-220 |
| 1.36 | Mir-20 |  |  | 2.12 | Mir-324-3p |
| 1.07 | Mir-31 |  |  | 2.66 | Mir-342 |
| 1.24 | Mir-200a |  |  | 3.09 | Mir-326 |
| 1.16 | Mir-17-5p |  |  | 2.19 | Mir-328 |
| 1.47 | Mir-141 |  |  | 2.19 | Mir-345 |
| 1.1 | Mir-21 |  |  | 2.18 | Mir-346 |
| 1.35 | Mir-106a |  |  | 4.99 | Mir-99a |
| 1.29 | Mir-122a |  |  | 2.29 | Mir-99b |
| 1.3 | Mir-100 |  |  |  |  |
| 1.28 | Mir-381 |  |  |  |  |
| 1.25 | Let-7e |  |  |  |  |
| 1.81 | Mir-133a |  |  |  |  |
| 1.18 | Mir-10a |  |  |  |  |
| 1.55 | Mir-22 |  |  |  |  |
| 1.02 | Mir-215 |  |  |  |  |

| Comparison of normal to uninvolved | | | | | | |
| --- | --- | --- | --- | --- | --- | --- |
| miRNAs up  regulated in normal | Fold change | miRNAs down  regulated in normal | Fold change | | Unchanged miRNAs | Fold change |
| Mir-186 | 2.29 |  |  | | Mir-150 | 1.18 |
| Mir-199a | 2.45 |  |  | | Mir-197 | 1.28 |
| Mir-22 | 2.22 |  |  | | Mir-99a | 1.027 |
| Mir-220 | 2.026 |  |  | |  |  |
| Mir-221 | 2.20 |  |  | |  |  |
| Mir-24-1 | 2.25 |  |  | |  |  |
| Mir-296 | 2.17 |  |  | |  |  |
| Mir-331 | 2.21 |  |  | |  |  |
| Mir-324-5p | 2.14 |  |  | |  |  |
| Mir-329 | 2.00 |  |  | |  |  |
| Mir-423 | 2.44 |  |  | |  |  |
| Comparison of uninvolved to psoriasis | | | | | | |
| miRNAs up  regulated in uninvolved | Fold change | miRNAs down  regulated in uninvolved | Fold change | Unchanged miRNAs | | Fold change |
| Mir-199a-1 | 2.73 | Mir-150 | 3.36 | Mir-197 | | 1.69 |
| Mir-210 | 2.82 |  |  | Mir-423 | | 1.67 |
| Mir-323 | 2.3 |  |  |  | |  |
| Mir-328 | 2.93 |  |  |  | |  |
| Mir-33 | 2.03 |  |  |  | |  |
| Mir-99a | 4.63 |  |  |  | |  |
