# Supplementary material for: MiRNA Expression in Psoriatic Skin: Reciprocal Regulation of hsa-miR-99a and IGF-1R
Source: PLoS One. 2011 Jun 7;6(6):e20916. doi: 10.1371/journal.pone.0020916 (PMC3110257; doi:10.1371/journal.pone.0020916)
Supplement: Table S2 — Comparison of normal versus psoriatic lesional skin. Total RNA from normal skin (n = 3), psoriatic lesional (n = 3), was isolated using Ambion mirVana™ miRNA Isolation Kit. Two µg of total RNA from each sample were labeled with the mirVana miRNA Labeling Kit (Applied Biosystems/Ambion Austin, TX 78744-1832, USA). The normal was labeled red and the psoriasis green. The fluorescently-labeled RNA samples were hybridized to an expression array as previously described [1]. The array was scanned and analyzed using Genepix pro 4000 b Axon and JMP statistic software. After background-subtracted, normalized spot intensities presented as loge of the scanned value, ratio calculated of N to P for individual spot is note (Each miRNA is represented on the CHIP either 4 or 8 times; the value is an average of all spots of the same miRNA). MiRNAs that were found to change more than two fold are highlighted as well as miRNAs that were further analysis. (DOC) [file pone.0020916.s004.doc]

**Table S2:** Comparison of miRNA expression profiles of normal versus psoriatic lesional skin. MiRNAs that were found to change more than two fold are highlighted, as well as miRNAs that were further analysis.

| miRNA name | normal (N) | psoriasis (P) | Ratio difference |
| --- | --- | --- | --- |
| Lodish_A01_1 let7a-1 | 7.50551428 | 7.25901309 | 1.279540706 |
| Lodish_A02_1 miR103-1 | 7.27796859 | 6.97386699 | 1.355406758 |
| Lodish_A03_1 let7b | 7.67472666 | 7.22436768 | 1.568875279 |
| Lodish_A04_1 miR105-1 | 7.13200316 | 6.84294512 | 1.33516922 |
| Lodish_A05_1 let7c | 7.67414197 | 7.27202079 | 1.494992485 |
| Lodish_A06_1 miR106a | 7.40975097 | 6.95795528 | 1.571130918 |
| Lodish_A07_1 let7d | 7.53518847 | 7.24814349 | 1.332484147 |
| Lodish_A08_1 miR106b | 7.19998806 | 7.00315671 | 1.217538685 |
| Lodish_A09_1 let7d* | 6.92106412 | 6.29032077 | 1.87900682 |
| Lodish_A10_1 miR107 | 7.06168859 | 6.85076284 | 1.234820666 |
| Lodish_A11_1 let7e | 7.54954684 | 7.31949325 | 1.25866746 |
| Lodish_A12_1 miR10a | 6.6852753 | 6.51789375 | 1.18220525 |
| Lodish_A13_1 let7f-1 | 7.18928153 | 7.20476627 | 0.984634532 |
| Lodish_A14_1 miR1-2 | 7.54912365 | 7.40121068 | 1.159411988 |
| Lodish_A15_1 let7f-2 | 7.46347584 | 7.38580874 | 1.080762813 |
| Lodish_A16_1 miR122a | 7.12714148 | 6.86982853 | 1.293449849 |
| Lodish_A17_1 let7g | 7.58398872 | 7.4028506 | 1.198580716 |
| Lodish_A18_1 miR124a-1 | 7.35142607 | 7.08928624 | 1.299708268 |
| Lodish_A19_1 let7i | 7.18975463 | 7.12778396 | 1.063931139 |
| Lodish_A20_1 miR125a | 7.03789193 | 6.77589647 | 1.299520643 |
| Lodish_A21_1 miR100 | 7.35937943 | 7.09592434 | 1.301418847 |
| Lodish_A22_1 miR125b-1 | 6.78270432 | 6.53886556 | 1.276138549 |
| Lodish_A23_1 miR101-1 | 7.3129617 | 7.14638001 | 1.181260029 |
| Lodish_A24_1 miR126 | 7.25201001 | 6.83880613 | 1.51165319 |
| Lodish_B01_1 miR126* | 7.16323993 | 6.72374173 | 1.551928265 |
| Lodish_B02_1 miR138-1 | 7.17067622 | 6.9809605 | 1.208905881 |
| Lodish_B03_1 miR127 | 7.13223931 | 6.68967532 | 1.556693452 |
| Lodish_B04_1 miR139 | 6.72847557 | 6.43217757 | 1.344870869 |
| Lodish_B05_1 miR128a | 7.22504277 | 6.62168893 | 1.828240155 |
| Lodish_B06_1 miR140 | 7.59630398 | 7.01034557 | 1.796712148 |
| Lodish_B07_1 miR129-2 | 7.09976201 | 6.58505398 | 1.673149921 |
| Lodish_B08_1 miR141 | 7.26483522 | 6.87432708 | 1.477731498 |
| Lodish_B09_1 miR130a | 7.60256434 | 7.24570079 | 1.428840891 |
| Lodish_B10_1 miR142-3p | 7.26898833 | 6.93995776 | 1.389620336 |
| Lodish_B11_1 miR130b | 7.62630297 | 7.3171833 | 1.362225378 |
| Lodish_B12_1 miR142-5p | 7.47660497 | 7.15330095 | 1.381685347 |
| Lodish_B13_1 miR132 | 7.38185215 | 7.0113132 | 1.448515082 |
| Lodish_B14_1 miR143 | 7.77416156 | 7.38980136 | 1.468674363 |
| Lodish_B15_1 miR133a-1 | 7.33 | 6.73633087 | 1.810619641 |
| Lodish_B16_1 miR144 | 7.33853605 | 7.08458868 | 1.289103957 |
| Lodish_B17_1 miR134 | 7.47342419 | 7.03470862 | 1.550714155 |
| Lodish_B18_1 miR145 | 7.48247383 | 7.23529377 | 1.280409643 |
| Lodish_B19_1 miR135a-1 | 7.18105684 | 6.81247867 | 1.445677645 |
| Lodish_B20_1 miR146 | 7.40333997 | 7.15865963 | 1.277212974 |
| Lodish_B21_1 miR136 | 7.34642355 | 7.02615289 | 1.377500548 |
| Lodish_B22_1 miR147 | 7.38947899 | 7.11579046 | 1.314805216 |
| Lodish_B23_1 miR137 | 7.40654862 | 7.17816671 | 1.256565129 |
| Lodish_B24_1 miR148a | 7.35663909 | 7.08547478 | 1.311490544 |
| Lodish_C01_1 miR149 | 7.44290749 | 6.24642032 | 3.308474378 |
| Lodish_C02_1 miR18 | 7.31592512 | 7.07976732 | 1.266374128 |
| Lodish_C03_1 miR150 | 7.63848446 | 6.18625854 | 4.272614436 |
| Lodish_C04_1 miR181a | 6.97704581 | 6.75698814 | 1.246148594 |
| Lodish_C05_1 miR151 | 7.57998609 | 7.16231763 | 1.518417175 |
| Lodish_C06_1 miR181b-1 | 6.92782377 | 6.59925322 | 1.388981229 |
| Lodish_C07_1 miR152 | 6.90326949 | 6.63175327 | 1.311952152 |
| Lodish_C08_1 miR182 | 7.44156246 | 6.9387687 | 1.653333842 |
| Lodish_C09_1 miR153-1 | 7.10077963 | 7.0137211 | 1.090960532 |
| Lodish_C10_1 miR182* | 7.06132559 | 6.62481993 | 1.547291 |
| Lodish_C11_1 miR154 | 7.36878491 | 6.80272053 | 1.761321501 |
| Lodish_C12_1 miR183 | 7.30074143 | 6.98565801 | 1.370373623 |
| Lodish_C13_1 miR155 | 7.32986629 | 7.09111891 | 1.269657755 |
| Lodish_C14_1 miR184 | 7.31947784 | 7.15535605 | 1.178357819 |
| Lodish_C15_1 miR15a | 7.35567144 | 7.19171845 | 1.178158929 |
| Lodish_C16_1 miR185 | 7.32225753 | 7.13127641 | 1.210436599 |
| Lodish_C17_1 miR15b | 7.12978396 | 6.6658245 | 1.590358496 |
| Lodish_C18_1 miR186 | 6.73308581 | 6.35416447 | 1.460708132 |
| Lodish_C19_1 miR16-1 | 7.22018293 | 6.92898308 | 1.338031963 |
| Lodish_C20_1 miR187 | 6.88518367 | 6.02958222 | 2.35278904 |
| Lodish_C21_1 miR17-3p | 7.39064021 | 7.10213007 | 1.334437881 |
| Lodish_C22_1 miR188 | 6.98963764 | 6.57394067 | 1.51542658 |
| Lodish_C23_1 miR17-5p | 7.41571675 | 7.25677645 | 1.17226796 |
| Lodish_C24_1 miR189 | 7.0043405 | 6.440605 | 1.757224367 |
| Lodish_D01_1 miR190 | 7.61505787 | 7.32957878 | 1.330399256 |
| Lodish_D02_1 miR199a-2 | 7.22403811 | 6.90749 | 1.372382266 |
| Lodish_D03_1 miR191 | 7.62344295 | 7.41992244 | 1.225710297 |
| Lodish_D04_1 miR19a | 7.40833519 | 7.14699254 | 1.298672579 |
| Lodish_D05_1 miR192 | 7.5263254 | 7.13968404 | 1.472028468 |
| Lodish_D06_1 miR20 | 6.95043625 | 6.64315121 | 1.35972849 |
| Lodish_D07_1 miR193 | 7.71497922 | 7.15216522 | 1.755605831 |
| Lodish_D08_1 miR200a | 6.94217731 | 6.72921863 | 1.237333524 |
| Lodish_D09_1 miR194-1 | 7.5157042 | 7.16535677 | 1.419560661 |
| Lodish_D10_1 miR200b | 7.15215293 | 6.90813912 | 1.276361957 |
| Lodish_D11_1 miR195 | 7.4049158 | 7.15550508 | 1.283268989 |
| Lodish_D12_1 miR201 | 7.36368998 | 7.09170066 | 1.312572983 |
| Lodish_D13_1 miR196a-1 | 7.37901609 | 7.04471328 | 1.396966095 |
| Lodish_D14_1 miR202 | 7.92533912 | 7.74196417 | 1.201264738 |
| Lodish_D15_1 miR197 | 7.11577299 | 5.90662221 | 3.350638015 |
| Lodish_D16_1 miR203 | 7.2802917 | 7.18307786 | 1.102096021 |
| Lodish_D17_1 miR198 | 7.60146786 | 7.33653965 | 1.303337406 |
| Lodish_D18_1 miR204 | 7.14046133 | 6.48293531 | 1.930011613 |
| Lodish_D19_1 miR199a*-1 | 7.23844059 | 6.86919986 | 1.44663581 |
| Lodish_D20_1 miR205 | 6.86607481 | 6.72116457 | 1.155935809 |
| Lodish_D21_1 miR199a*-2 | 7.05693002 | 6.73793306 | 1.375747143 |
| Lodish_D22_1 miR206 | 7.48605512 | 7.32831115 | 1.170866379 |
| Lodish_D23_1 miR199a-1 | 7.24629622 | 6.72864268 | 1.678085466 |
| Lodish_D24_1 miR207 | 6.79140991 | 6.39480397 | 1.486769938 |
| Lodish_E01_1 miR208 | 7.16992503 | 6.96807147 | 1.223668801 |
| Lodish_E02_1 miR22 | 7.10224596 | 6.65788002 | 1.559501065 |
| Lodish_E03_1 miR21 | 7.17402701 | 7.07915439 | 1.09951879 |
| Lodish_E04_1 miR220 | 7.43526755 | 6.28017136 | 3.174328742 |
| Lodish_E05_1 miR210 | 7.94544161 | 7.32535454 | 1.859089906 |
| Lodish_E06_1 miR221 | 7.25339556 | 6.7294748 | 1.688635422 |
| Lodish_E07_1 miR211 | 6.85110068 | 6.34920823 | 1.651844347 |
| Lodish_E08_1 miR222 | 6.97907933 | 6.66635777 | 1.367140811 |
| Lodish_E09_1 miR212 | 7.52496651 | 6.92468173 | 1.822637777 |
| Lodish_E10_1 miR223 | 7.58964005 | 7.29186403 | 1.346860084 |
| Lodish_E11_1 miR213 | 6.94450475 | 6.57173331 | 1.451752489 |
| Lodish_E12_1 miR224 | 6.77960409 | 6.68344964 | 1.100929089 |
| Lodish_E13_1 miR214 | 7.33255802 | 7.0869234 | 1.278432375 |
| Lodish_E14_1 miR23a | 7.0503033 | 6.59117541 | 1.5826931 |
| Lodish_E15_1 miR215 | 7.49674562 | 7.47320067 | 1.023824321 |
| Lodish_E16_1 miR23b | 7.18238426 | 6.85884809 | 1.382006143 |
| Lodish_E17_1 miR216 | 6.83514227 | 6.5799114 | 1.290759584 |
| Lodish_E18_1 miR24-1 | 7.37618151 | 7.25047372 | 1.133950768 |
| Lodish_E19_1 miR217 | 7.3337733 | 7.32192568 | 1.011918081 |
| Lodish_E20_1 miR25 | 6.95879608 | 6.53362287 | 1.529855383 |
| Lodish_E21_1 miR218-1 | 7.15191401 | 6.6844851 | 1.595885748 |
| Lodish_E22_1 miR26a-1 | 7.77211382 | 7.24239727 | 1.698450814 |
| Lodish_E23_1 miR219-1 | 7.09045479 | 6.83172217 | 1.295287425 |
| Lodish_E24_1 miR27a | 7.56338634 | 7.02233108 | 1.717818651 |
| Lodish_F01_1 miR28 | 7.50830652 | 7.17068435 | 1.401610833 |
| Lodish_F02_1 miR299 | 7.08474637 | 6.81165817 | 1.314016136 |
| Lodish_F03_1 miR290 | 7.41110144 | 6.9172999 | 1.638533345 |
| Lodish_F04_1 miR29b | 7.2076331 | 6.86364024 | 1.410568564 |
| Lodish_F05_1 miR291-3p | 7.31200768 | 6.86073049 | 1.570316498 |
| Lodish_F06_1 miR300 | 7.22200652 | 6.92221074 | 1.349583168 |
| Lodish_F07_1 miR291-5p | 7.52064361 | 7.11665431 | 1.497787921 |
| Lodish_F08_1 miR301 | 7.18162244 | 7.05846295 | 1.1310648 |
| Lodish_F09_1 miR292-3p | 7.25722651 | 7.06880263 | 1.207345176 |
| Lodish_F10_1 miR302a | 7.2959208 | 7.02508605 | 1.3110584 |
| Lodish_F11_1 miR292-5p | 7.35611809 | 7.00647773 | 1.418557287 |
| Lodish_F12_1 miR302b* | 7.06385595 | 6.88458755 | 1.196341799 |
| Lodish_F13_1 miR293 | 7.31636379 | 7.04291223 | 1.314493684 |
| Lodish_F14_1 miR302c | 7.07223906 | 6.85591218 | 1.241508137 |
| Lodish_F15_1 miR294 | 7.20497657 | 6.87858276 | 1.385961067 |
| Lodish_F16_1 miR302c* | 7.32524887 | 6.75822838 | 1.763006323 |
| Lodish_F17_1 miR295 | 7.20277633 | 6.98438508 | 1.244073716 |
| Lodish_F18_1 miR30a-3p | 7.13320208 | 6.65228595 | 1.617555615 |
| Lodish_F19_1 miR296 | 7.14998263 | 6.68617604 | 1.590115396 |
| Lodish_F20_1 miR30a-5p | 7.66138751 | 7.35451348 | 1.359169743 |
| Lodish_F21_1 miR297-1 | 7.51658451 | 7.03825184 | 1.613382118 |
| Lodish_F22_1 miR30b | 7.28689624 | 7.08480069 | 1.223964952 |
| Lodish_F23_1 miR298 | 7.49180215 | 7.12869827 | 1.437785209 |
| Lodish_F24_1 miR31 | 7.45239077 | 7.38776489 | 1.066759854 |
| Lodish_G01_1 miR32 | 7.28411863 | 7.1626238 | 1.129183528 |
| Lodish_G02_1 miR331 | 6.65217832 | 6.29431033 | 1.430276797 |
| Lodish_G03_1 miR320 | 7.97410714 | 7.77966184 | 1.21463704 |
| Lodish_G04_1 miR337 | 7.06438952 | 6.57342831 | 1.633885973 |
| Lodish_G05_1 miR322 | 7.13552822 | 6.79512132 | 1.40551938 |
| Lodish_G06_1 miR338 | 7.11894552 | 6.78754378 | 1.392919271 |
| Lodish_G07_1 miR323 | 7.18385697 | 6.78345369 | 1.492426442 |
| Lodish_G08_1 miR339 | 6.82159748 | 6.2886814 | 1.703893762 |
| Lodish_G09_1 miR324-3p | 7.25267733 | 6.50102946 | 2.120491433 |
| Lodish_G10_1 miR340 | 6.7710339 | 6.53432663 | 1.267070154 |
| Lodish_G11_1 miR324-5p | 7.01407968 | 6.63305647 | 1.463781579 |
| Lodish_G12_1 miR341 | 6.9328453 | 6.26869946 | 1.942830324 |
| Lodish_G13_1 miR325 | 7.03030445 | 6.66980733 | 1.434042128 |
| Lodish_G14_1 miR342 | 7.39970118 | 6.42050044 | 2.662327499 |
| Lodish_G15_1 miR326 | 7.27378421 | 6.14569442 | 3.08974879 |
| Lodish_G16_1 miR344 | 6.67110638 | 6.29626927 | 1.45475443 |
| Lodish_G17_1 miR328 | 6.79501988 | 6.01196527 | 2.188146 |
| Lodish_G18_1 miR345 | 7.06233069 | 6.27868424 | 2.189441416 |
| Lodish_G19_1 miR329 | 6.67230473 | 6.31749491 | 1.425909449 |
| Lodish_G20_1 miR346 | 7.23265417 | 6.45053754 | 2.186094525 |
| Lodish_G21_1 miR33 | 7.29012585 | 6.69758578 | 1.808576497 |
| Lodish_G22_1 miR34a | 6.7979145 | 6.60798478 | 1.209164615 |
| Lodish_G23_1 miR330 | 7.04032496 | 6.81376761 | 1.25427454 |
| Lodish_G24_1 miR34c | 7.16248256 | 6.89474751 | 1.307000804 |
| Lodish_H01_1 miR350 | 7.27587263 | 6.7719404 | 1.655217185 |
| Lodish_H02_1 miR376b | 7.2975546 | 7.08335514 | 1.238869735 |
| Lodish_H03_1 miR351 | 7.41630322 | 6.92479581 | 1.634778645 |
| Lodish_H04_1 miR380 | 7.30382 | 7.0444217 | 1.296149959 |
| Lodish_H05_1 miR367 | 7.28610095 | 7.15527726 | 1.139766811 |
| Lodish_H06_1 miR409 | 7.14613518 | 6.7695309 | 1.457327502 |
| Lodish_H07_1 miR368 | 7.7317117 | 7.35627596 | 1.455625551 |
| Lodish_H08_1 miR410 | 7.4250934 | 7.23784404 | 1.205927958 |
| Lodish_H09_1 miR369 | 7.45168621 | 7.2141708 | 1.268094538 |
| Lodish_H10_1 miR411 | 7.33222825 | 7.06788208 | 1.302579032 |
| Lodish_H11_1 miR370 | 7.42735471 | 6.9356941 | 1.635029112 |
| Lodish_H12_1 miR412 | 7.19350715 | 6.66411013 | 1.697908195 |
| Lodish_H13_1 miR371 | 7.35447111 | 6.93672274 | 1.518538516 |
| Lodish_H14_1 miR7-1 | 7.49471409 | 7.17217925 | 1.380622991 |
| Lodish_H15_1 miR372 | 7.32381301 | 7.0649265 | 1.295486772 |
| Lodish_H16_1 miR9-1 | 7.22202594 | 6.93883088 | 1.327364052 |
| Lodish_H17_1 miR373 | 7.29992319 | 7.04571257 | 1.289443358 |
| Lodish_H18_1 miR9-1* | 7.79113548 | 7.43548755 | 1.427105019 |
| Lodish_H19_1 miR373* | 7.37792103 | 6.97184906 | 1.500910569 |
| Lodish_H20_1 miR92-1 | 7.4368483 | 6.84352552 | 1.809992642 |
| Lodish_H21_1 miR374 | 7.54609216 | 7.23415481 | 1.366069106 |
| Lodish_H22_1 miR93 | 7.42578605 | 7.11230107 | 1.368184912 |
| Lodish_H23_1 miR376a | 7.53913775 | 7.23745162 | 1.352136765 |
| Lodish_H24_1 miR95 | 7.71303044 | 7.08688105 | 1.870394535 |
| Lodish_I01_1 miR96 | 6.89207257 | 6.58483829 | 1.359659472 |
| Lodish_I02_1 miR98 | 7.10322643 | 6.92400759 | 1.19628251 |
| Lodish_I03_1 miR99a | 7.57682995 | 5.96920508 | 4.990943001 |
| Lodish_I04_1 miR99b | 6.90495843 | 6.07510876 | 2.292974012 |
| Lodish_I05_1 miR335 | 7.45566117 | 7.36729321 | 1.092390004 |
| Lodish_I06_1 miR361 | 7.51522394 | 7.22348472 | 1.338753852 |
| Lodish_I07_1 miR381 | 7.27041555 | 7.02294973 | 1.280775585 |
| Lodish_I08_1 miR382 | 7.24202884 | 6.96377063 | 1.320827204 |
| Lodish_I09_1 miR424 | 6.88682069 | 6.75947341 | 1.135811394 |
| Lodish_J05_1 miR383 | 7.201792 | 6.940596 | 1.298482143 |
| Lodish_J06_1 miR384 | 7.86197245 | 7.47671946 | 1.469986166 |
| Lodish_J07_1 miR422a | 7.59525494 | 7.26695685 | 1.388602839 |
| Lodish_J08_1 miR423 | 7.88577545 | 7.53384486 | 1.421809832 |
| Lodish_J09_1 miR425 | 7.45886243 | 7.15598017 | 1.353755064 |
