# Supplementary material for: MiRNA Expression in Psoriatic Skin: Reciprocal Regulation of hsa-miR-99a and IGF-1R
Source: PLoS One. 2011 Jun 7;6(6):e20916. doi: 10.1371/journal.pone.0020916 (PMC3110257; doi:10.1371/journal.pone.0020916)
Supplement: Table S5 — Patient characteristics. (DOC) [file pone.0020916.s007.doc]

**Table S5: Patient** characteristics:

| **number** | **sex** | **age** | **Biopsy location** | **Clinical diagnosis** | **Pathologic diagnosis** |  |
| --- | --- | --- | --- | --- | --- | --- |
| 1 | F | 73 | upper right limb | Psoriasis Vulgaris | Psoriasis |  |
| 2 | F |  |  | Psoriasis Vulgaris | Cronic psoriasiform spongiotic dermatitis | Excluded |
| 3 | M | 61 |  | Psoriasis Vulgaris | Psoriasis |  |
| 4 | F | 87 | lower left limb | Psoriasis Vulgaris | Psoriasis |  |
| 5 | F | 26 | upper right limb | Psoriasis Vulgaris | Psoriasis |  |
| 6 | M | 63 | upper left limb | Psoriasis Vulgaris | Psoriasiform dermatitis | Excluded |
| 7 | M | 65 | upper left limb | Psoriasis Vulgaris |  |  |
| 8 | F | 36 | lower right limb | Psoriasis Vulgaris | Psoriasiform dermatitis | Excluded |
| 9 | M | 49 | upper right limb | Psoriasis Vulgaris | Psoriasis |  |
| 10 | M | 51 | lower right limb | Psoriasis Vulgaris | Psoriasis |  |
| 11 | M | 47 | lower right limb | Psoriasis Vulgaris | Psoriasis |  |
| 12 | F | 48 | upper left limb | Psoriasis Vulgaris | Psoriasis |  |
| 13 | M | 49 | upper right limb | Psoriasis Vulgaris | Psoriasis |  |
| 14 | F | 56 | upper left limb | Psoriasis Vulgaris | Psoriasiform dermatitis | Excluded |
| 15 | M | 73 | lower left limb | Psoriasis Vulgaris | Psoriasis |  |
| 16 | M | 28 | upper right limb | Psoriasis Vulgaris | Psoriasis |  |
| 17 | F |  | upper left limb | Psoriasis Vulgaris | psoriasiform dermatitis | Excluded |
| 18 | M | 44 | lower left limb | Psoriasis Vulgaris | Psoriasis |  |
